# Epidemiology of Human Metapneumovirus Infection in a Community Setting, Seattle, Washington, USA

**DOI:** 10.1093/infdis/jiaf142

**Published:** 2025-07-16

**Authors:** Annalyse Elias-Warren, Julia C Bennett, Chidozie D Iwu, Lea M Starita, Jeremy Stone, Ben Capodanno, Robin Prentice, Peter D Han, Zack Acker, Sally B Grindstaff, David Reinhart, Jennifer K Logue, Caitlin R Wolf, Michael Boeckh, Kevin Kong, Hong Xie, Geon Kim, Alexander L Greninger, Amanda C Perofsky, Cécile Viboud, Timothy M Uyeki, Janet A Englund, Pavitra Roychoudhury, Helen Y Chu

**Affiliations:** Department of Medicine, University of Washington, Seattle, Washington, USA; Department of Epidemiology, University of Washington, Seattle, Washington, USA; Department of Medicine, University of Washington, Seattle, Washington, USA; Department of Epidemiology, University of Washington, Seattle, Washington, USA; Department of Medicine, University of Washington, Seattle, Washington, USA; Department of Epidemiology, University of Washington, Seattle, Washington, USA; Brotman Baty Institute for Precision Medicine, Seattle, Washington, USA; Department of Genome Sciences, University of Washington, Seattle, Washington, USA; Brotman Baty Institute for Precision Medicine, Seattle, Washington, USA; Brotman Baty Institute for Precision Medicine, Seattle, Washington, USA; Brotman Baty Institute for Precision Medicine, Seattle, Washington, USA; Brotman Baty Institute for Precision Medicine, Seattle, Washington, USA; Department of Genome Sciences, University of Washington, Seattle, Washington, USA; Brotman Baty Institute for Precision Medicine, Seattle, Washington, USA; Brotman Baty Institute for Precision Medicine, Seattle, Washington, USA; Brotman Baty Institute for Precision Medicine, Seattle, Washington, USA; Department of Medicine, University of Washington, Seattle, Washington, USA; Department of Medicine, University of Washington, Seattle, Washington, USA; Department of Medicine, University of Washington, Seattle, Washington, USA; Brotman Baty Institute for Precision Medicine, Seattle, Washington, USA; Vaccine and Infectious Disease Division, Fred Hutchinson Cancer Center, Seattle, Washington, USA; Department of Laboratory Medicine and Pathology, University of Washington, Seattle, Washington, USA; Department of Laboratory Medicine and Pathology, University of Washington, Seattle, Washington, USA; Department of Laboratory Medicine and Pathology, University of Washington, Seattle, Washington, USA; Department of Laboratory Medicine and Pathology, University of Washington, Seattle, Washington, USA; Brotman Baty Institute for Precision Medicine, Seattle, Washington, USA; Fogarty International Center, National Institutes of Health, Bethesda, Maryland, USA; Fogarty International Center, National Institutes of Health, Bethesda, Maryland, USA; Influenza Division, Centers for Disease Control and Prevention, Atlanta, Georgia, USA; Seattle Children's Research Institute, Seattle, Washington, USA; Vaccine and Infectious Disease Division, Fred Hutchinson Cancer Center, Seattle, Washington, USA; Department of Laboratory Medicine and Pathology, University of Washington, Seattle, Washington, USA; Department of Medicine, University of Washington, Seattle, Washington, USA

**Keywords:** human metapneumovirus, community setting, genomic epidemiology, clinical epidemiology, geospatial percent positivity

## Abstract

**Background:**

The clinical and genomic epidemiology of human metapneumovirus (hMPV) infections in community settings is not well understood.

**Methods:**

From 2018 to 2022, individuals with respiratory symptoms were recruited and enrolled from the greater Seattle, Washington community in the United States. Residual clinical specimens from individuals presenting with respiratory symptoms were additionally collected. Specimens were tested for hMPV by reverse-transcription polymerase chain reaction, with whole genome sequencing performed on a subset (209/1002).

**Results:**

hMPV positivity was higher among clinical specimens (835/21 539 [3.9%]) compared to community specimens (167/28 348 [0.6%]). Children aged 0–4 years had the highest percent positivity across both clinical and community settings (497/10 213 [4.9%] and 28/1640 [1.7%], respectively). In multivariate analysis, a household income of ≤US$100 000 (adjusted odds ratio [aOR], 1.72 [95% confidence interval {CI}, 1.07–2.85]), and recent international travel (aOR, 6.51 [95% CI, 3.11–12.22]) were associated with hMPV positivity. A subset of 209 of 1002 samples (21%) was sequenced; the distribution of subtypes A2b, A2c, B1, and B2 were similar across both community and clinical settings, with an increase in the proportion of subtype B1 after the start of the pandemic.

**Conclusions:**

Risk factors of testing positive for hMPV in a community setting included lower household income and recent international travel. Co-circulation of hMPV subtypes was observed across community and clinical settings.

Human metapneumovirus (hMPV) is responsible for a substantial burden of medically attended acute respiratory illness (ARI), particularly in younger children and older adults [1–5]. Characteristics of hMPV infection in community-dwelling children and adults are not well described, though it is likely that infections in the community lead to onward transmission to vulnerable populations.

Several hMPV vaccines are in development [6–8]. Characterizing risk factors and demographics associated with hMPV in a community setting, specifically across all age groups, may inform vaccine policy recommendations and help establish vaccine priority groups if vaccines become available. Baseline data prior to vaccine implementation in community settings are also needed for comparison to postvaccine epidemiology.

Previous literature has suggested differential disease severity by hMPV subtype [9, 10]. Compared to other respiratory viruses, there is little characterization of hMPV subtype prevalence and limited information on heterogeneity in subtype circulation based on temporal, geographic, and clinical characteristics. Genomic data may support vaccine antigen selection and allow for monitoring of viral evolution with use of therapeutics or vaccines.

Finally, the impact of the coronavirus disease 2019 (COVID-19) pandemic on hMPV community transmission is not well characterized; studies to date have primarily focused on clinical cases [11–13]. Analyzing trends of hMPV circulation before and after the COVID-19 pandemic may help quantify its impact on subtype prevalence. Similarly, mapping the geospatial distribution of hMPV infections both before and after the COVID-19 pandemic may allow for further understanding of the impact of pandemic mitigation measures on hMPV.

The objective of this study was to describe the sociodemographic and clinical factors associated with hMPV detection in individuals in community settings, and to characterize the genomic epidemiology of hMPV before and during the COVID-19 pandemic, in both community and clinical settings.

## METHODS

This was a secondary analysis using data from the Seattle Flu Study (SFS) [14]. SFS was an observational year-round study of individuals with respiratory illnesses across the greater Seattle area from 2018 to 2022, including King, Snohomish, Pierce, Skagit, and Island counties in Washington state in the United States (US). Individuals with a reported home address outside of the surveillance counties were excluded from this analysis.

SFS included 2 main sources of data: (1) community-based surveillance through in-person enrollment at community sites or via online enrollment and home-based specimen collection; and (2) medical record data and residual specimens from individuals tested as part of routine clinical care at regional hospitals.

### Community Surveillance

Participants were recruited and enrolled at in-person study kiosks across various community settings, including childcare facilities, workplaces, university campuses, and transit stations, as well as remotely online through the study website [15]. In-person testing transitioned to fully remote home-based specimen collection during the COVID-19 pandemic (Supplementary Figure 1). Remote online enrollment prior to the pandemic was conducted through the “Swab and Send” substudy, while enrollment during the COVID-19 pandemic was conducted through the Seattle Coronavirus Assessment Network (SCAN) substudy. Clinical and demographic data were self-reported at time of enrollment through an electronic questionnaire, either administered in-person or self-administered online. Convenience sampling was used to enroll eligible community participants.

Individuals experiencing 2 or more new or worsening respiratory symptoms at the time of enrollment were eligible for community surveillance inclusion; symptoms were required to be present within the past 7 days to be eligible. Symptoms of respiratory illness included feeling feverish, rhinorrhea, headache, rash, cough, fatigue, diarrhea, muscle or body aches, sore throat, increased trouble breathing, nausea or vomiting, ear pain or discharge, and for SCAN, loss of taste and smell [14]. Individuals were able to participate repeatedly in SCAN, Swab and Send, and kiosks, at least 2 weeks apart. Childcare kiosks included weekly repeat sampling of enrolled children <18 years of age, regardless of symptoms. Asymptomatic individuals were allowed to participate in SCAN and the childcare studies but not Swab and Send or kiosks; due to the variation in asymptomatic enrollment practices across substudies, asymptomatic individuals across all studies were excluded from this analysis. To address repeating sampling from the same individual, this analysis only included the first hMPV-positive swab collected from an individual or the last hMPV-negative swab collected if an individual had no hMPV-positive swabs during the study period.

### Residual Clinical Data and Specimens

Clinical specimens from patients residing in the surveillance area who presented for a medically attended ARI at urgent care clinics, outpatient clinics, and hospitals affiliated with Seattle Children's Hospital and the University of Washington during the surveillance period (2018–2022) were tested retrospectively. Samples from children and adults in both inpatient and outpatient settings were included. Clinical specimens were only included in this analysis if the patient resided in 1 of the surveillance counties where community sampling occurred. ARI visits were identified using J-codes and *International Classification of Diseases, 10th Revision* codes. Seattle Children's Hospital locations are estimated to account for 80% of children's emergency department visits and 85% of children's ARI visits for King County, Washington, making it a representative sample of the community it serves. Seattle Children's Hospital receives patients from across Washington state and the surrounding states of Alaska, Idaho, and Montana. Limited demographic information including age, sex, and census tract was collected through electronic medical record data extraction.

### Laboratory Testing and Sequencing

All community and clinical nasal swabs were tested for 26 respiratory pathogens, including hMPV, via reverse-transcription polymerase chain reaction (RT-PCR) (Thermo Fisher Open Array platform) [14–16]. Relative cycle threshold (Crt) values generated during testing were considered a proxy for semiquantitative viral load. hMPV-positive specimens with Crt values <20 were eligible for whole genome sequencing. A total of 91 community and 657 clinical specimens with Crt <20 were eligible for sequencing. A subset of clinical specimens was randomly selected for an approximate 2:1 match to community specimens based on respiratory season, resulting in 244 samples eligible for sequencing: 91 (100%) community and 153 (23.3%) clinical samples. Community specimens collected during the 2018–2019 respiratory season were unable to be located for sequencing, resulting in 71 community specimens. Sequencing was performed using a hybridization probe capture–based approach and consensus genome assembly was performed using a custom bioinformatic pipeline (Supplementary Material). All available hMPV sequences in GenBank with month and year date information (185 sequences as of October 2024) were downloaded and included in the phylogenetic tree.

### Statistical Methods

We characterized demographic characteristics of participants who tested positive and negative for hMPV from community surveillance. Detailed symptom profiles and percent positivity by age were described for hMPV-positive individuals in clinical and community settings. Binomial logistic regression was used to estimate odds of testing positive for hMPV in the community by participant characteristics. Known predictors of hMPV-related hospitalization including age (0–4, 5–17, 18–49, and ≥50) and sex at birth were included. Other possible risk factors evaluated included income level (≤US$100 000, US$100 001–US$150 000, and >US$150 000), household size (lives alone, 2 people, 3–4 people, and ≥5 people), domestic travel outside of Washington state in the 2 weeks prior to testing, and international travel in the 2 weeks prior to testing. In univariate and multivariate analysis, a complete case analysis was used in relation to all the variables included in the model.

Counts were used to describe hMPV subtypes by time (respiratory season and before vs during the COVID-19 pandemic), age group, and setting (clinical vs community). Phylogenetic trees including study sequences and all available hMPV sequences from GenBank with known collection date were created using Nextstrain, Augur, Auspice, and the ggtree package in R [17, 18].

Percent positivity for hMPV was mapped by Public Use Microdata Areas (PUMAs) for each respiratory virus season (2018–2019, 2019–2020, 2020–2021, and 2021–2022) to visualize variation across the city of Seattle and over time. PUMAs divide state counties into geographic regions containing at least 100 000 individuals. The geospatial analysis was restricted to the city of Seattle as sampling density was greatest among participants living in the city of Seattle. Maps were generated using survey and sryvr packages in R [19, 20]. Statistical reweighting to account for community sampling bias was not performed.

R Studio version 4.4.1 was used for all statistical analyses [21].

### Human Subjects Protection

This study was approved by the University of Washington Human Subjects Institutional Review Board. All participants completed informed consent (or parental or guardian consent for participants <18 years of age at enrollment).

## RESULTS

### hMPV Surveillance

**Figure 1.**
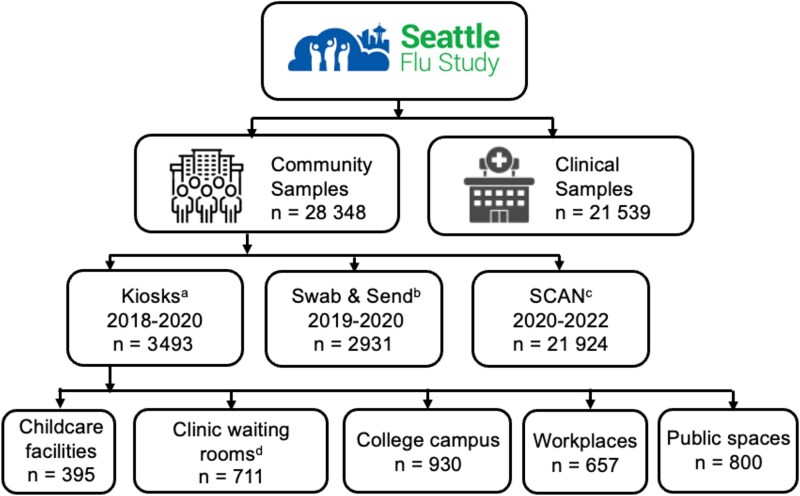
Seattle Flu Study enrollment strategies and nasal swab collection methods. ^a^Kiosk swabs were collected by research/clinical staff between 2018 and 2019, and self-collected by the participants in 2020 under supervision due to coronavirus disease 2019 precautions. ^b^Swab & Send swabs were self-collected at home unsupervised and mailed to the laboratory. ^c^Seattle Coronavirus Assessment Network (SCAN) swabs were self-collected unsupervised and mailed to the laboratory. ^d^These individuals are in clinical wait rooms for a multitude of reasons other than seeking care for influenza-like illness.

From November 2018 to July 2022, 74 987 specimens were collected and tested for hMPV; 49 617 were from individuals in community settings and 25 370 were from clinical settings. A total of 25 100 samples were excluded from this analysis—19 962 due to repeat testing and 5138 due to asymptomatic status. This resulted in a total of 49 887 specimens included in our analysis: 21 539 specimens from a clinical setting and 28 348 specimens from a community setting (Figure 1). A total of 1002 (1002/49 887 [2.0%]) were hMPV-positive, of which 167 (167/28 348 [0.6%]) were from community settings and 835 (835/21 539 [3.9%]) were from clinical settings. The majority of community enrollment was through home-based remote sample collection via the SCAN substudy (online enrollment during the COVID-19 pandemic; n = 21 924) or the Swab and Send substudy (online enrollment prior to the COVID-19 pandemic; n = 2931) with fewer from in-person kiosks (n = 3493) (Figure 1). Percent positivity of hMPV in community settings was highest among individuals enrolled through in-person kiosks (72/3493 [2.1%]) and Swab and Send (62/2931 [2.1%]) and was lower for individuals enrolled through SCAN (33/21 924 [0.2%]), reflecting enrollment periods for each substudy prior to and during the pandemic, respectively (Table 1). Residual clinical specimens came from Seattle Children's Hospital locations (n = 17 516), University of Washington Hospital locations (n = 3967), and Seattle Children's outpatient clinics (n = 253). The hMPV percent positivity in clinical settings was highest among individuals presenting for care at Seattle Children's Hospital locations (693/17 516 [4.0%]) and University of Washington Hospital locations (144/3967 [3.6%]) and was lower for individuals presenting for care at Seattle Children's outpatient clinics (5/253 [2.0%]).

**Table 1.** Demographic Characteristics of Individuals Tested for Human Metapneumovirus in Community Settings (N = 28 348)

Characteristic	Community hMPV-Positives (n = 167)	Community hMPV-Negatives (n = 28 181)	Total (n = 28 348)
Age, y			
0–4	28 (16)	1612 (6)	1640 (6)
5–17	12 (7)	2112 (7)	2124 (7)
18–49	96 (58)	19 048 (68)	19 144 (68)
≥50	20 (12)	5124 (18)	5144 (18)
Missing	11 (7)	285 (1)	296 (1)
Sex			
Female	104 (62)	16 391 (58)	16 495 (58)
Male	52 (31)	11 137 (40)	11 189 (40)
Unknown	11 (7)	653 (2)	664 (2)
Race			
American Indian/Alaska Native	0 (0)	154 (1)	154 (1)
Asian	18 (11)	4370 (15)	4388 (15)
Black/African American	2 (1)	811 (3)	813 (3)
Native Hawaiian	1 (1)	268 (1)	269 (1)
Multiracial	6 (4)	1626 (6)	1632 (6)
White	79 (47)	16 235 (58)	16 314 (58)
Other	7 (4)	1271 (4)	1278 (4)
Missing	54 (32)	3446 (12)	3500 (12)
Ethnicity			
Hispanic/Latino	16 (10)	2410 (9)	2426 (9)
Not Hispanic/Latino	100 (60)	22 733 (81)	22 833 (80)
Unknown	51 (30)	3038 (11)	3089 (11)
Household income level, US$			
≤100 000	56 (33)	7673 (27)	7729 (27)
100 001–150 000	12 (7)	4085 (15)	4097 (15)
>150 000	28 (17)	7018 (25)	7046 (25)
Unknown	71 (43)	9405 (33)	9476 (33)
Household size			
Lives alone	11 (7)	2692 (10)	2703 (9)
2 people	33 (20)	7195 (25)	7228 (25)
3–4 people	34 (20)	11 220 (40)	11 254 (40)
≥5 people	36 (22)	4144 (15)	4180 (15)
Unknown	53 (32)	2930 (10)	2983 (11)
Comorbidities^a^			
None	82 (49)	15 434 (55)	15 516 (55)
At least 1 comorbidity	24 (14)	8847 (31)	8871 (31)
Unknown	61 (37)	3900 (14)	3961 (14)
International travel in the 2 wk prior to testing	11 (7)	531 (2)	541 (2)
Domestic travel out of state in the 2 wk prior to testing	10 (6)	3474 (12)	3484 (12)
Sought medical care^b^			
Did not seek care	81 (48)	22 034 (78)	22 115 (78)
Sought care	11 (7)	1528 (5)	1539 (5)
Missing	75 (45)	4619 (16)	4694 (17)
COVID-19 pandemic^c^			
Pre-COVID-19 (Nov 2018–Feb 2020)	114 (68)	5032 (18)	5146 (18)
COVID-19 (March 2020–July 2022)	53 (32)	23 149 (82)	23 202 (82)
Respiratory season^d^			
2018–2019	44 (26)	1440 (5)	1484 (5)
2019–2020	85 (51)	5746 (20)	5831 (21)
2020–2021	10 (6)	217 356 (62)	17 366 (61)
2021–2022	26 (16)	3359 (12)	3385 (12)
2022–2023^b^	2 (1)	71 (<1)	73 (<1)
Unknown	0 (0)	209 (1)	209 (1)
Surveillance county			
King	146 (87)	26 607 (94)	26 753 (94)
Snohomish	5 (3)	395 (1)	400 (1)
Pierce	0 (0)	164 (1)	164 (1)
Island	0 (0)	2 (<1)	2 (<1)
Skagit	0 (0)	2 (<1)	2 (<1)
Other^e^	16 (10)	1011 (4)	1027 (4)

Abbreviations: COVID-19, coronavirus disease 2019; hMPV, human metapneumovirus; US$, United States dollars.

^a^Comorbidities include any of the following: allergies, asthma, bronchitis, chronic obstructive pulmonary disease, cystic fibrosis, pulmonary fibrosis, neurologic conditions, down syndrome, cerebrovascular conditions, high blood pressure, hypertension, cardiovascular disease, blood conditions like thalassemia or sickle cell anemia, diabetes, obesity, liver disease, kidney disease, immunocompromised conditions, immunosuppressed conditions, and cancer.

^b^Clinical care seeking for their illness at the time of testing, including urgent care, telehealth, primary care, or hospitalization.

^c^Anything collected on March 1st, 2020 or after was considered collected after the start of the pandemic.

^d^Respiratory seasons were defined as starting in July and finishing in June of the following year.

^e^County variable was created based on census tract. Community samples with a census outside of the surveillance counties were assumed to spend enough time in the surveillance counties to be included in analysis.

Median Crt value was higher (corresponding to lower viral load) among positive specimens from community settings (18.7 [range: 2.6–27.5]) compared to clinical settings (14.2 [range: 5.3–27.9]). Approximately a quarter of hMPV-positive cases (n = 250) had 1 or more other virus detected by RT-PCR. The most common coinfection with hMPV was rhinovirus (92/1002 [9.2%]), followed by influenza (65/1002 [6.5%]) and adenovirus (48/1002 [4.8%]). Other pathogens co-detected with hMPV included human non–severe acute respiratory syndrome coronavirus 2 (SARS-CoV-2) coronaviruses, enteroviruses, parainfluenza viruses, respiratory syncytial virus, and SARS-CoV-2. Across all age groups who tested positive for hMPV, children aged 0–4 and 5–17 years had the highest frequency of coinfections (161/525 [30.7%] and 63/199 [31.6%], respectively). Clinical specimens had a higher frequency of coinfection compared to community specimens that tested positive for hMPV (225/835 [27.0%] and 25/167 [15.0%], respectively).

Most community and clinical positives were collected prior to the COVID-19 pandemic on 1 March 2020 (114/167 [68%] and 626/835 [75%], respectively). Community and clinical hMPV percent positivity was highest during the 2018–2019 respiratory season (44/1484 [3.0%] and 419/8074 [5.2%], respectively) and the 2019–2020 season (85/5831 [1.5%] and 259/5525 [4.7%]). Community and clinical percent hMPV positivity declined during the 2020–2021 (10/17 366 [0.1%] and 3/3059 [0.1%]) and 2021–2022 (26/3385 [0.8%] and 124/4425 [2.8%]) seasons, corresponding to mitigation measures implemented during the COVID-19 pandemic.

**Figure 2.**
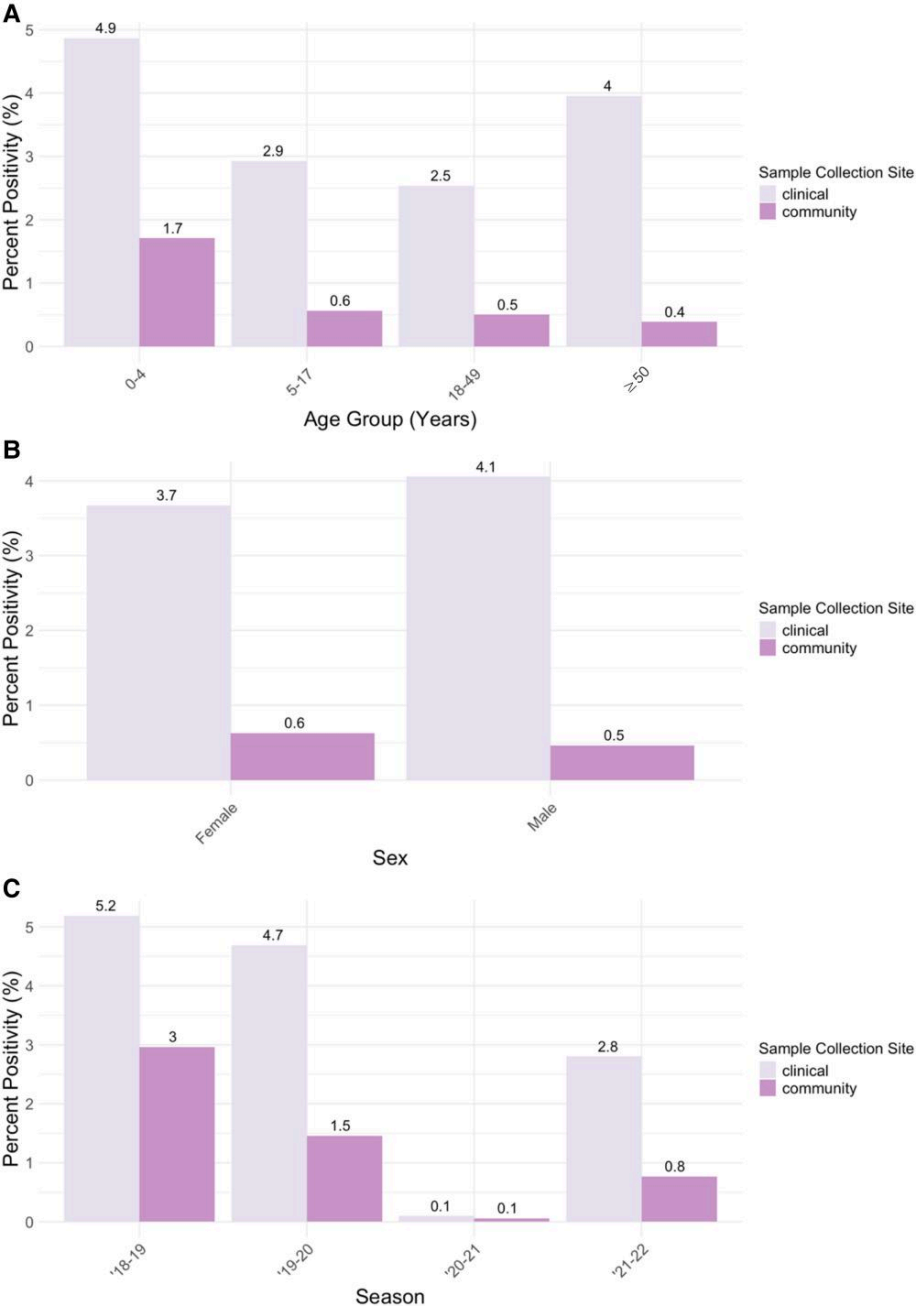
Percent positivity by collection site and age group (*A*), collection site and sex (*B*), and collection site and season (*C*).

Percent positivity was higher among clinical versus community specimens for every age group (Figure 2). For both clinical and community specimens, percent positivity was highest among children aged 0–4 years (497/10 213 [4.9%] and 28/1640 [1.7%], respectively). For community specimens, percent positivity was lower and similar for all other age groups (<1%). For clinical specimens, percent positivity was also relatively high among adults aged ≥50 years (72/1821 [4.0%]) and lower for individuals aged 5–49 years (between 2.5% and 2.9%).

### Clinical Presentation and Risk Factors in Community Settings

**Figure 3.**
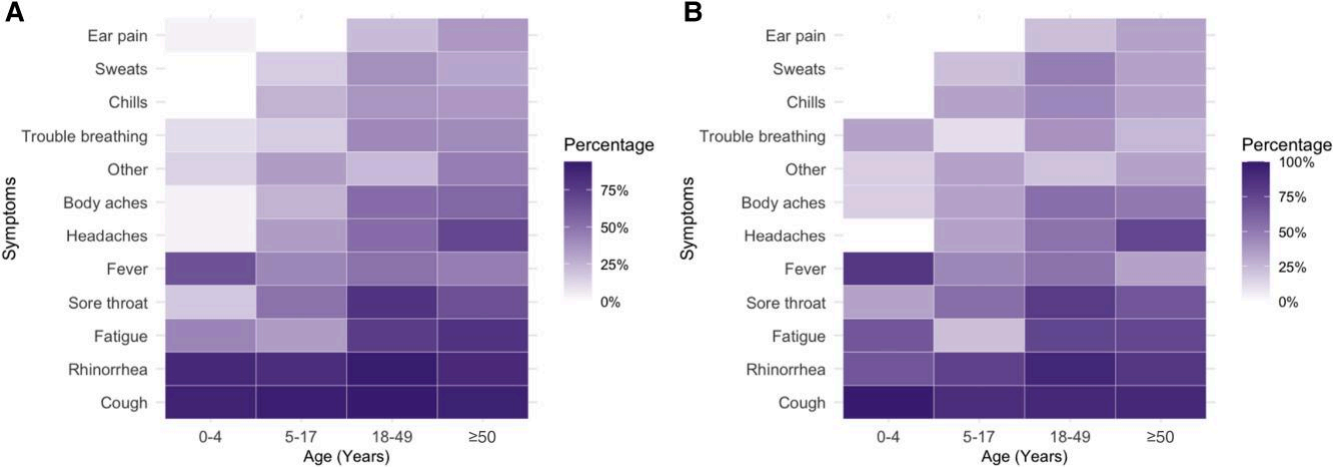
Percentage of reported symptoms by age for human metapneumovirus community positive cases for all positives (n = 156; 11 excluded due to missing age profiles) (*A*) and positives within 4 days of symptom onset (n = 85; 11 excluded due to missing age profiles, 71 excluded for being outside 4 days of symptom onset) (*B*).

Among individuals testing positive for hMPV in community settings, cough (range: 89.3%–93.8%) and rhinorrhea (range: 83.3%–92.7%) were commonly reported across all age groups (Figure 3, Supplementary Table 3). Fever was more common among children 0–4 years of age (18/28 [64.3%]) than age groups ≥5 years (range, 41.7%–51.3%). Fatigue, body aches, headaches, and sore throat were more common among adults (range: 75.0%–80.0%, 53.1%–55.0%, 53.1%–70.0%, and 65.0%–80.2%, respectively) than children <18 years of age (range: 33.3%–42.9%, 3.6%–25.0%, 3.6%–33.3%, and 17.9%–50.0%). Symptom frequency was similar among hMPV-positive specimens collected from individuals in community settings with and without a low Crt value (Crt <15; Supplementary Table 2). A small proportion (11/167 [6.6%]) of individuals with hMPV in community settings reported care-seeking for their illness, but none were hospitalized (Table 1). Among the 11 community individuals who reported care-seeking for their illness, 9 were between 18 and 49 years of age and 2 were ≥50 years of age.

In multivariate analysis of risk factors for hMPV positivity among community cases, a household income of ≤US$100 000 (adjusted odds ratio [aOR], 1.72 [95% confidence interval {CI}, 1.07–2.85]) and international travel within the 2 weeks prior to testing (aOR, 6.51 [95% CI, 3.11–12.22]) were significantly associated with hMPV positivity in community settings (Table 2). Canada was the most common location of international travel among community hMPV-positive cases (5/11 [45%]). Male sex was associated with a lower odds of testing positive for hMPV in community settings (aOR, 0.58 [95% CI, .36–.92]).

**Table 2.** Results of Univariable and Multivariable Models Assessing Risk Factors for Testing Positive for Human Metapneumovirus in a Community Setting (N = 18 402^a^)

Variables	hMPV Positive (n = 90)	hMPV Negative (n = 18 312)	Unadjusted OR (95% CI); *P* Value	Adjusted OR (95% CI); *P* Value
Age group, y (18–49 reference)				
0–4	5	1054	0.91 (.32–2.05); .84	1.21 (.42–2.83); .68
5–17	6	1320	0.87 (.34–1.86); .75	0.95 (.36–2.08); .90
≥50	13	3240	0.77 (.41–1.35); .40	0.74 (.39–1.30); .32
Sex (female reference)				
Male sex	24	7127	0.57 (.35–.90); .02^b^	0.58 (.36–.92); .03^b^
Income (>US$150 000 reference)				
≤$100 000	52	7435	1.85 (1.17–3.01); .01^b^	1.72 (1.07–2.85); .03^b^
$100 001–$150 000	12	4004	0.79 (.39–1.54); .50	0.81 (.39–1.57); .54
Household size (2 people reference)				
Lives alone	8	1803	0.81 (.34–1.69); .59	0.74 (.31–1.56); .46
3–4 people	27	8372	0.59 (.35–.99); .05	0.63 (.36–1.08); .09
≥5 people	26	2858	1.66 (.97–2.82); .06	1.65 (.94–2.86); .07
International travel in the 2 wk prior to testing	10	384	5.84 (2.82–10.81); <.0001^b^	6.51 (3.11–12.22); <.0001^b^
Domestic travel out of state in the 2 wk prior to testing	8	2623	0.58 (.26–1.13); .15	0.53 (.23–1.03); .09

Abbreviations: CI, confidence interval; hMPV, human metapneumovirus; OR, odds ratio; US$, United States dollars.

^a^Sample size for univariate and multivariate risk factor analysis was restricted to specimens with data for all risk factors evaluated in this table.

^b^Represents a statistically significant *P* value (<.05).

### Subtype Distributions in Clinical Versus Community Specimens

**Figure 4.**
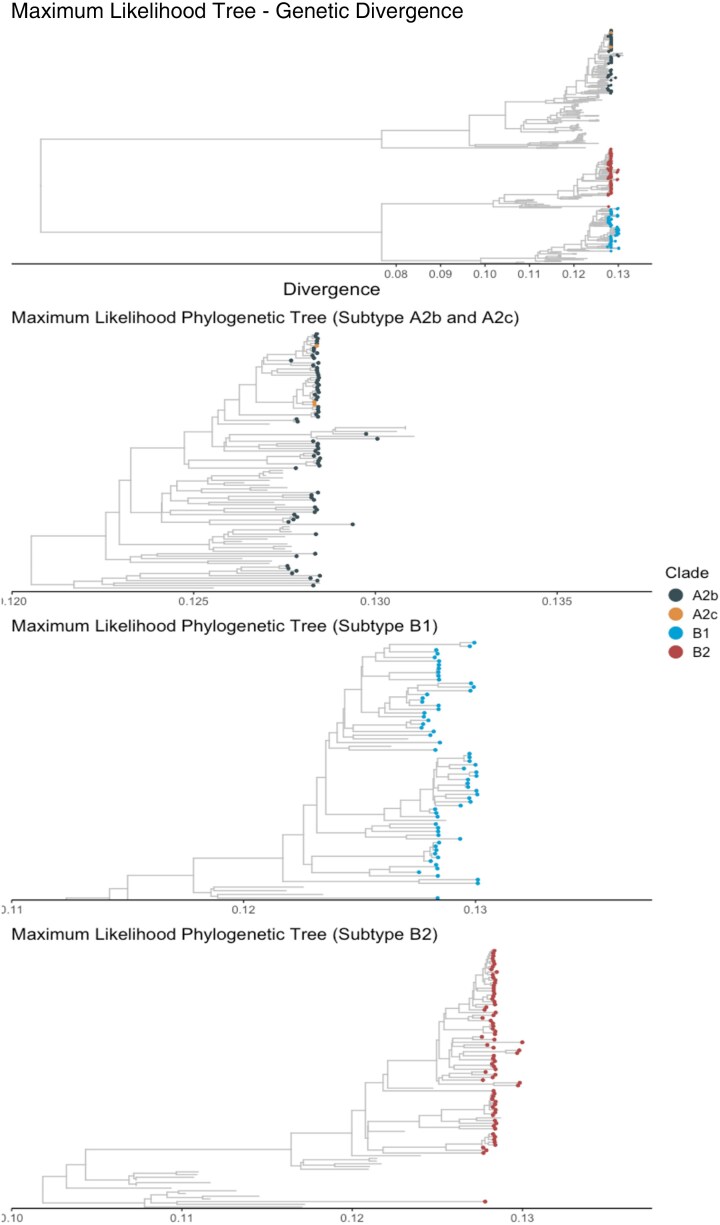
Maximum likelihood phylogenetic trees of sequenced samples (colored tips) compared to contextual human metapneumovirus (hMPV) sequences from GenBank (gray) identifying the 3 main circulating hMPV subtypes (A2b, B1, and B2, zoomed in view in the 3 lower panels).

**Figure 5.**
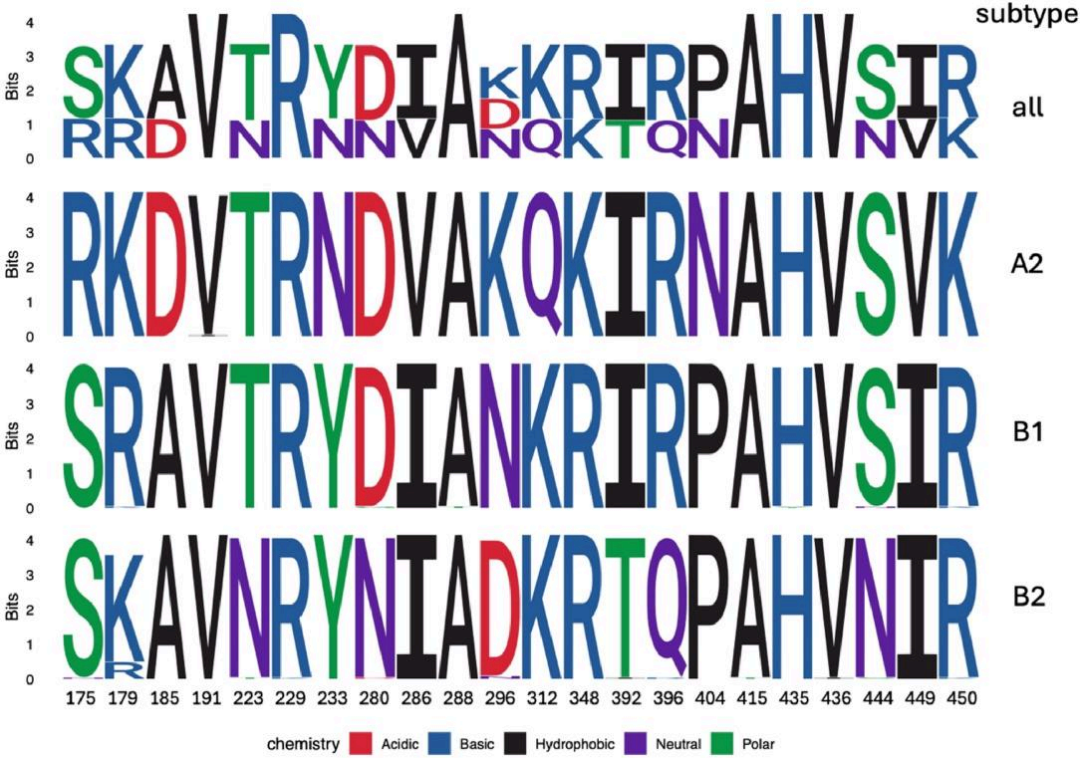
Sequence logo plot illustrating amino acid variation within the F1 subunit of the RSV F gene overall across all sequences (n = 237), and within each subtype (n = 82, 75, and 80 sequences for subtype A2, B1, and B2 respectively). At each position, the total height of the stack is measured in bits and reflects the information content—i.e., how conserved that position is across sequences as measured by Shannon entropy. Taller stacks indicate higher conservation. The height of each individual letter within a stack represents the relative frequency of that amino acid at that site. Only positions with observed variation are shown (see Supplemental Tables 4 and 5).

High-quality genomes were obtained from 210 of the 244 (86.1%) selected samples. During sequencing, 1 hMPV RT-PCR–positive sample was determined to be an enterovirus and was reclassified as a negative hMPV swab due to this finding for all analyses in this paper. Of the resulting 209 high-quality hMPV genomes, 138 were from clinical settings and 71 were from community settings. More than half of clinical specimens (103/138 [75%]) and community specimens (46/71 [65%]) were collected prior to the COVID-19 pandemic. Sequences from the 2018–2019 respiratory virus season are all from clinical settings, whereas remaining seasons (2019–2020, 2020–2021, and 2021–2022) include sequences from both clinical and community settings. Phylogenetic trees include all study sequences and 185 contextual sequences from GenBank. Of the 209 sequences, 72 (34%) were subtype A2b, 3 (1%) were A2c, 62 (30%) were B1, and 72 (34%) were B2. Subtypes A1 and A2a were not identified in our study sequences (Figure 4). The fusion protein (F) gene was highly conserved among our sequenced samples with 97% mean pairwise amino acid identity across all identified subtypes, and 99.7%–99.9% within each subtype (Supplementary Table 4). Much of the amino acid variation was subtype-specific (Figure 5), and the majority of variable sites were in the F1 domain (Supplementary Tables 4 and 5), consistent with previous studies [22].

**Figure 6.**
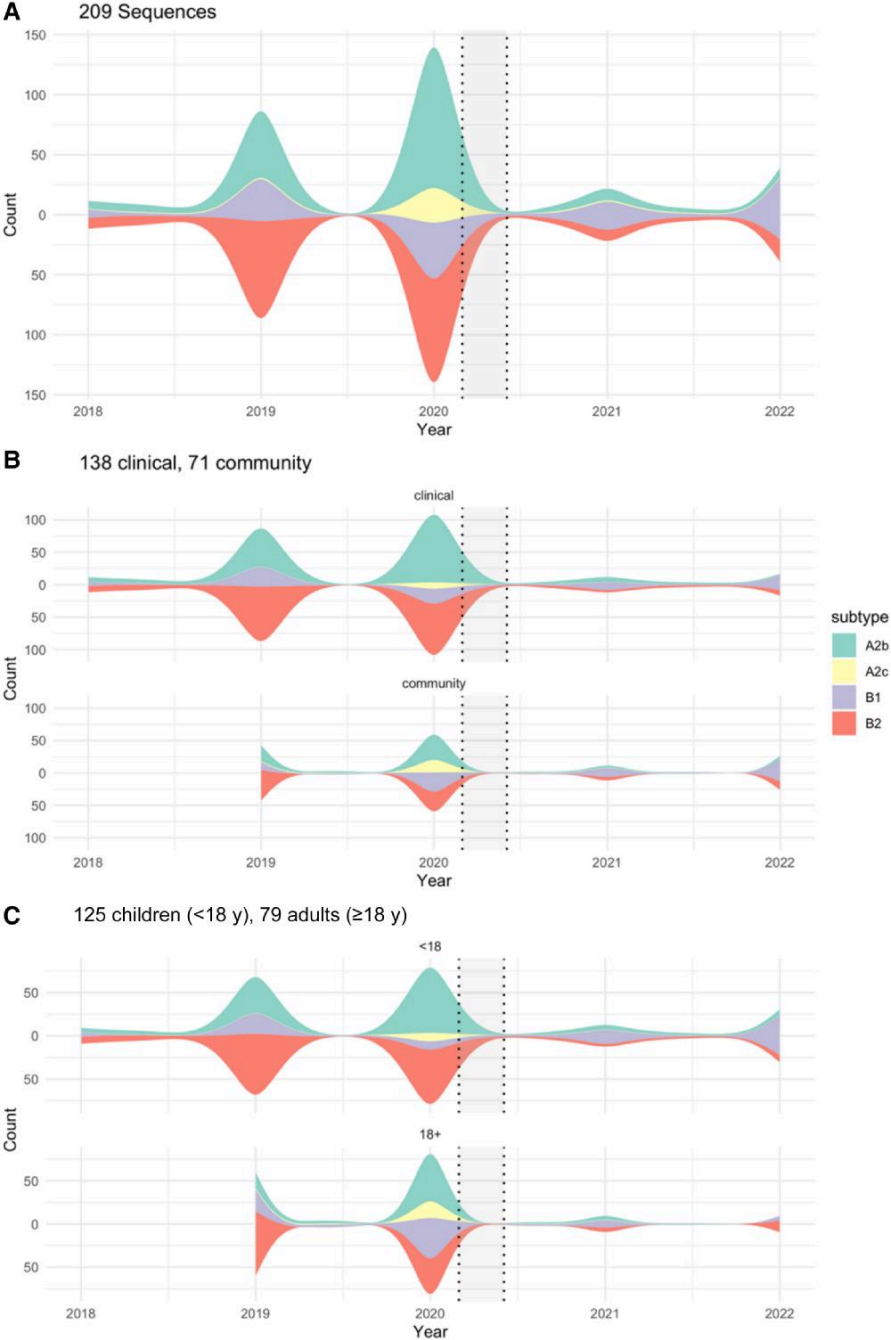
Human metapneumovirus subtypes observed from November 2018 to July 2022 in the Seattle Flu Study overall (*A*), by community vs clinical settings (*B*), and by age (*C*). Total number of sequences is the sum of counts in a year above and below zero on the y-axis and data are smoothed over time. In *C*, 204 sequences were included as 5 sequences were missing age data.

Prior to the COVID-19 pandemic, subtypes A2b and B2 were most prevalent in our sample (50/149 [34%] and 61/149 [41%], respectively) and among children <18 years of age (32/86 [37%] and 37/86 [43%], respectively; Figure 6), whereas subtypes B1 and B2 were most prevalent among adults (18/58 [31%] and 23/58 [40%], respectively). After the start of the COVID-19 pandemic, subtype B1 was most prevalent (27/60 [45%]), followed by A2b (22/60 [37%]) and B2 (11/60 [18%]). There were no significant differences in the distribution of subtypes observed in community versus clinical settings (Table 3).

**Table 3.** Two-Sample Z-Test for Proportions to Evaluate Human Metapneumovirus Subtypes by Sample Type (Community Versus Clinical)

Subtype	Community Sequences (n = 71) No. (%)	Clinical Sequences (n = 138) No. (%)	*P* Value (α = .05)
A2b	22 (31)	50 (36)	.55
A2c	2 (3)	1 (<1)	.55
B1	25 (35)	37 (27)	.27
B2	22 (31)	50 (36)	.55

### Percent Positivity by Public Use Microdata Area

**Figure 7.**
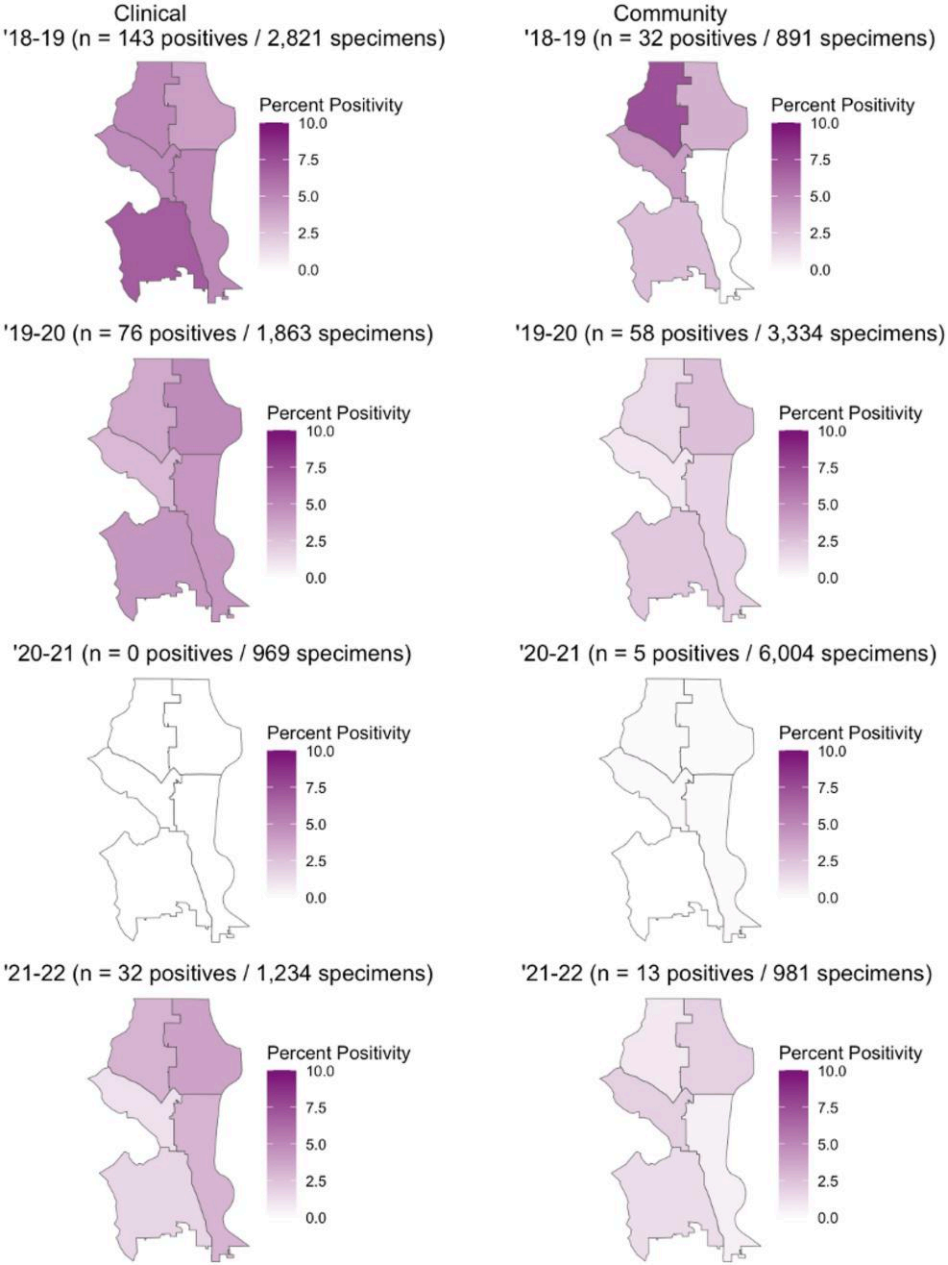
Human metapneumovirus percent positivity by public use microdata area in Seattle, Washington, across respiratory season and sampling method.

Among the 28 348 community and 21 539 clinical specimens tested for hMPV, the geospatial analysis included 11 210 (39.5%) community and 6887 (32.0%) clinical specimens from individuals residing in the city of Seattle. There was differential percent positivity by geography (PUMA) in Seattle, and this varied between clinical and community settings (Figure 7, Supplementary Table 6). In the 2018–2019 respiratory season, clinical specimens had the highest hMPV percent positivity in southwest Seattle (36/524 [6.9%]), whereas percent positivity among community specimens was highest in northwest Seattle (9/122 [7.4%]) and lowest in southeast Seattle (0/79 [0%]) (Figure 7). In 2019–2020 across 4 of 5 PUMAs, percent positivity among community specimens decreased (absolute percent positivity declined between 0.4% and 5.9% compared to 2018–2019) and among clinical specimens to a lesser degree (between 0.7% and 2.5%). From 2018–2019 to 2019–2020 the community percent positivity in the southeast PUMA slightly increased (0% to 1.8%) and the clinical percent positivity in the northeast PUMA slightly increased (4.0% to 4.9%). Percent positivity was 0% for clinical specimens and near 0% for community specimens (percent positivity range: 0.0%–0.2%) in the 2020–2021 season across all PUMAs. During 2021–2022, percent positivity remained low but slightly increased among community specimens (absolute percent positivity increased between 0.5% and 1.8% compared to 2020–2021) and increased to a slightly larger degree among clinical specimens (between 1.2% and 3.9%) across all PUMAs.

**Figure 8.**
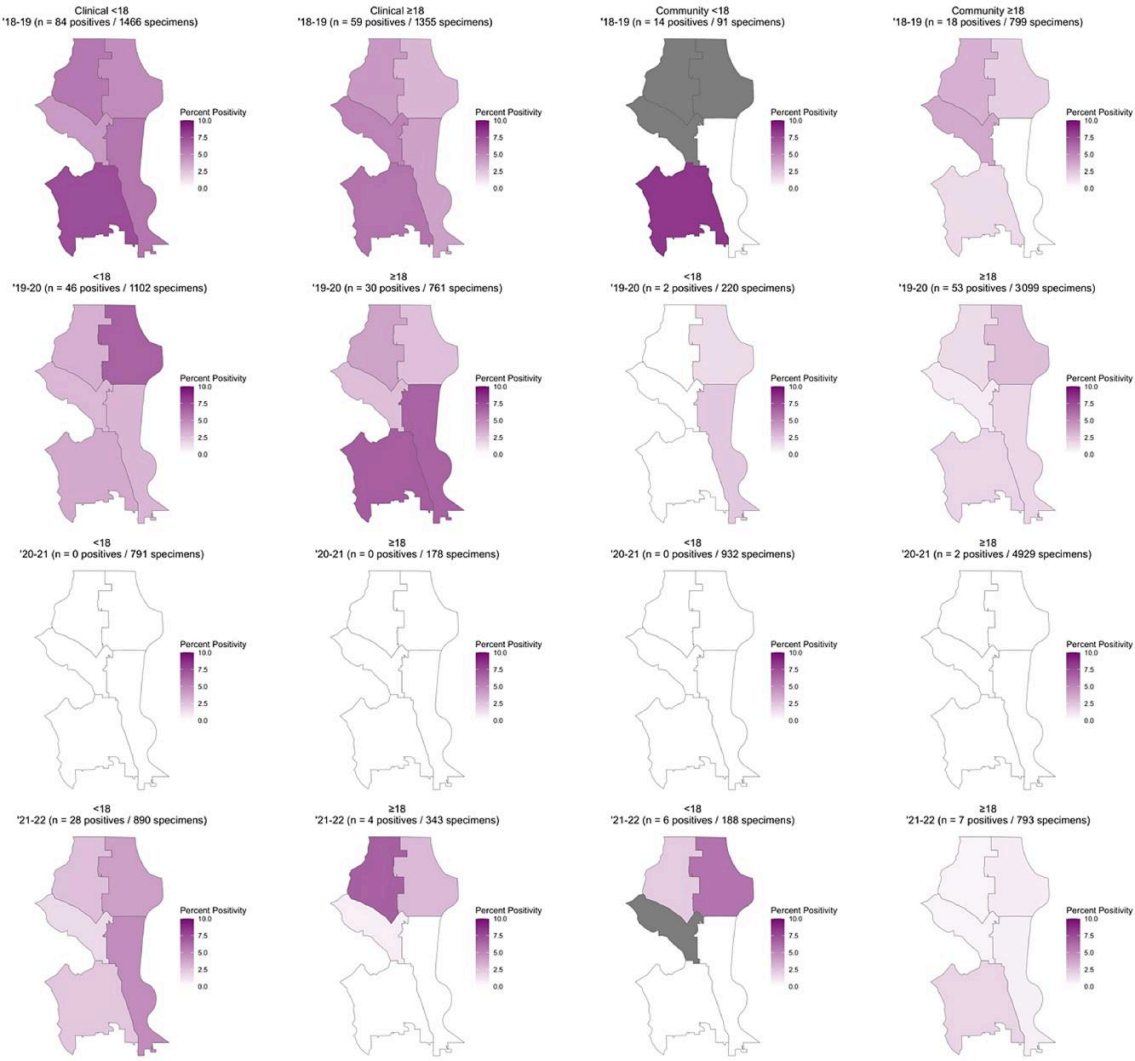
Human metapneumovirus percent positivity by Public Use Microdata Area (PUMA) in Seattle, Washington, across respiratory season, sampling method, and age group (<18 years old and ≥18 years old). Gray areas represent a percent positivity that is >10%. See Supplementary Tables 7 and 8 for PUMA-specific counts.

The geospatial analysis was further stratified by age group (<18 years vs ≥18 years) to evaluate the influence of age group on percent positivity (Figure 8, Supplementary Tables 7 and 8). This analysis included 11 051 (39.0%) community and 6886 (32.0%) clinical specimens and displayed differential percent positivity by geography and age across clinical and community settings (Figure 8). During the 2018–2019 season, children <18 years of age had the highest percent positivity in both clinical settings (25/336 [7.4%] for children in southwest Seattle) and community settings (6/28 [21.4%] for children in northeast Seattle). During the 2019–2020 season, the highest percent positivity was observed in adult populations in both clinical settings (6/89 [6.7%] for adults in southwest Seattle) and community settings (19/699 [2.7%] for adults in northeast Seattle). The 2020–2021 season had zero or near zero percent positivity across all age groups in clinical and community settings. In the 2021–2022 season, the highest percent positivity for clinical samples was observed in adults (2/30 [6.7%] for adults in northwest Seattle), whereas the highest percent positivity for community samples was observed in children (3/28 [10.7%] for kids in western Seattle).

## DISCUSSION

In a study of individuals with respiratory illness in community settings, we noted risk factors for hMPV positivity, including an income of ≤US$100 000 and recent international travel. Male sex was associated with a lower odds of testing positive for hMPV in a community setting. We found that the COVID-19 pandemic had an impact on both frequency and diversity of circulating hMPV subtypes. Most hMPV cases in our study occurred prior to the pandemic and consistent with other studies, we observed a decline in percent positivity from the 2019–2020 to the 2020–2021 respiratory season [23].

Our study documented hMPV symptom profiles across age groups among community illnesses. Among all age groups, a very high percentage of individuals reported cough and rhinorrhea. Notably, fever was not as commonly reported in adults compared to children 0–4 years of age, while the proportion reporting headache was higher in older age groups. In another study from New York including community and clinical participants, younger adults reported a higher percentage of hoarseness, whereas older adults reported a higher percentage of dyspnea and wheezing [5]. We did not find differences in proportions reporting sore throat or trouble breathing across adult age groups. Very few adult community cases reported care-seeking for their hMPV illness, which is consistent with prior literature on respiratory viral infection in healthy adults who often have milder illness and do not seek care [24, 25].

Low socioeconomic status indicators, like household income, has previously been associated with respiratory virus test positivity and severe outcomes such as hospitalization; we found that a household income below our surveillance area's median income appears to be a risk factor for testing positive for hMPV in a community setting [26, 27]. In our model, we found that household income ≤US$100 000 was associated with testing positive for hMPV in a community setting. In King County, where the majority of our specimens are from, the median income is approximately US$122 000 [28]. International travel has also previously been described as a risk factor for influenza and other respiratory viruses, and this appears to also be a risk factor for testing positive for hMPV in a community setting [29]. In our study, community hMPV-positive samples from individuals reporting international travel were primarily detected from November 2019 to March 2020. However, the number of sequenced samples from this subset was too small (4 sequenced samples out of 11 hMPV positive) to draw significant conclusions about importation of strains through travel. While the 4 sequences were more closely related to other study samples than to GenBank sequences, this could be the result of unsampled genetic diversity outside the study. Male sex was associated with a lower odds of testing positive for hMPV in community settings, which may be related to health-seeking behaviors and research participation behaviors between the 2 sexes.

Percent positivity was higher overall in clinical settings, and patterns by age differed between clinical versus community cases. While children 0–4 years old had the highest percent positivity in both settings, patterns for other age groups diverged. In clinical settings, percent positivity followed a “U”-shaped pattern (highest in children <5 years of age and adults ≥50 years of age and lower in older children and younger adults), whereas in community settings percent positivity for all other age groups other than children 0–4 years of age was lower and similar (∼0.5% across the study period). Our findings are in line with previous studies which have found that those at highest risk for hospitalization or clinical care for their hMPV infection are typically young children or older adults [4, 30].

Our study described hMPV subtype prevalence in community and clinical settings, across age groups, and over time, contributing substantially to the data available on hMPV subtypes beyond prior studies, which primarily focused on hMPV subtype prevalence in clinical settings [31–34]. We did not find subtype A1 in our study samples, consistent with previous studies, which noted that this subtype has not been detected after 2006 [31]. It is unclear why subtype A2a was not found in our samples, but it echoes a study from Kenya that had similar findings with no detection of A1 or A2a [35]. It remains unclear whether there is a relationship between subtype and symptom severity or clinical outcomes like hospitalization, with conflicting conclusions from previous studies [9, 10]. Additional studies are needed to further evaluate if there is a relationship between infection severity and subtype or genotype as our study found no significant difference in the proportion of subtypes across clinical and community settings. Co-circulation of multiple subtypes across clinical and community settings suggests that it may be strategic to focus vaccine development on targeting regions conserved across hMPV subtypes along with continued genomic surveillance of hMPV.

Our study also showed a shift in the distribution of circulating hMPV subtypes following implementation of nonpharmaceutical interventions to reduce transmission of SARS-CoV-2 in 2020, similar to previous studies on other respiratory viruses like influenza [36]. It is unclear if B1 will continue to be the dominant subtype in the postpandemic era and monitoring should continue. While a 2021 study from Israel found a shift from A2b to B1 in their hospitalized population [11], another study from South Korea found that subtypes A2b and B2 circulated after the COVID-19 pandemic [12], and a study from Spain found that subtype A2c was dominant postpandemic [32].

Percent positivity for clinical and community specimens varied across Seattle by PUMA. Percent positivity of hMPV decreased in the middle of the 2019–2020 respiratory season and slowly increased toward the end of the 2020–2021 respiratory season, which is roughly in line with the implementation and easing of social distancing measures put into place during the COVID-19 pandemic. Among clinical specimens, percent positivity was highest in southern Seattle PUMAs prepandemic, whereas percent positivity was highest in the northwest PUMA for community specimens. According to the City of Seattle's Racial and Social Equity Composite Index, which combines measures of race, ethnicity, socioeconomic disadvantage, and health disadvantage, there are a greater number of sociodemographic disadvantaged census tracts in southern Seattle PUMAs compared to northern Seattle PUMAs [37]. It is possible that differences in sociodemographic status between northern and southern Seattle PUMAs contributed to higher observed percent positivity among clinical specimens from individuals residing in southern Seattle PUMAs. This adds to existing literature that suggests socioeconomic status is associated with respiratory virus percent positivity and severe respiratory infection outcomes, such as hospitalization [38, 39]. Interventions to reduce respiratory infection burden should therefore continue to be targeted toward areas with lower socioeconomic status and historically higher rates of respiratory illness. Certain PUMAs had small sample sizes for specific years, which made some estimates unstable. The COVID-19 pandemic interrupted transmission during our surveillance period; future studies should explore the relationship between hMPV positivity and geographic area over a longer period of time as some of the differences in geospatial distribution of hMPV may have been due to changes from the COVID-19 pandemic, rather than differential care-seeking in individuals from different regions. Also, we were unable to account for bias resulting from uneven sampling across PUMAs for community specimens, which limits these findings.

Limitations of our study include missing information for certain variables including income (11%) and household size (16%), which limits generalizability of our risk factor analysis results. Second, clinical specimens had limited demographic information available to the study, so we were unable to evaluate differences in risk factors for hMPV infection between community and clinical settings. Third, we were only able to sequence a subset of samples, and it is possible that the samples selected do not truly reflect hMPV subtype distribution, particularly for low viral load infections. This may impact the generalizability of our genomic findings. We were also unable to sequence community samples collected during the 2018–2019 season, which restricted our ability to compare community subtype distribution before and after the COVID-19 pandemic. Fourth, observed percent positivity during the 2019–2020 respiratory virus season reflects both prepandemic months and pandemic months (beginning March 2020), when hMPV infections declined to near zero. This likely impacts our percent positivity estimates during the 2019–2020 respiratory season since this season captures prepandemic and pandemic months. Fifth, convenience sampling was used to capture community specimens and our results for community settings may not be generalizable to the wider Seattle metropolitan area population, including older age groups and certain geographic regions that were underrepresented. Finally, there was a change in swab procedures during the course of the study due to the interruption of the COVID-19 pandemic. Although swabbing procedures changed from staff-collected swabs to remote self-collected swabs, self-collected swabs have been found in prior studies to be comparable to staff-collected nasal swabs in terms of sensitivity for detection of SARS-CoV-2; this has not been evaluated for hMPV, which is a limitation of the study design [40].

## CONCLUSIONS

Our study describes the epidemiology and genomic diversity of community and clinical hMPV infections. A household income of ≤US$100 000 and recent international travel was associated with higher odds of infection with hMPV in the community, and we observed the highest clinical hMPV percent positivity in geographic areas with low socioeconomic status. Our genomic analysis suggested that multiple subtypes of hMPV circulated prepandemic and that the pandemic changed the frequency of circulating hMPV in both community and clinical settings. A vaccine targeting regions conserved across subtypes should be considered to reduce the burden of hMPV. Future studies should continue to evaluate risk factors for hMPV infection in community settings to further our understanding of nonclinical hMPV infection and guide future vaccine policy and implementation.

## Supplementary Material

jiaf142_Supplementary_Data

## Supplementary Data

Supplementary materials are available at *The Journal of Infectious Diseases* online (http://jid.oxfordjournals.org/). Supplementary materials consist of data provided by the author that are published to benefit the reader. The posted materials are not copyedited. The contents of all supplementary data are the sole responsibility of the authors. Questions or messages regarding errors should be addressed to the author.

Sequence logo plots in Figure 6 were generated in R using the ggseqlogo package (https://doi.org/10.1093/bioinformatics/btx469) with the y-axis representing information content in bits based on Shannon entropy (https://pubmed.ncbi.nlm.nih.gov/2172928/).

## Notes

***Acknowledgments.*** We thank all of the Seattle Flu Study staff, managers, and participants for their collaboration and help with our research study.

***Data availability.*** Genomic data can be found in BioProject PRJNA1029161; all other data are not publicly available.

***Financial support.*** Data collected as part of the Seattle Flu Study were funded by Gates Ventures and the US Centers for Disease Control and Prevention (CDC).

***Supplement sponsorship.*** This article appears as part of the supplement “Human Metapneumovirus Disease Burden in Older Adults and Implications for Adult Vaccination Strategies,” sponsored by AstraZeneca.
