# Supplementary material for: Epidemiology of Human Metapneumovirus Infection in a Community Setting, Seattle, Washington, USA
Source: J Infect Dis. 2025 Jul 16;232(Suppl 1):S78–92. doi: 10.1093/infdis/jiaf142 (PMC12265066; doi:10.1093/infdis/jiaf142)
Supplement: jiaf142_Supplementary_Data [file jiaf142_supplementary_data.docx]

**Title: Epidemiology of Human Metapneumovirus Infection in a Community Setting, Seattle, WA, USA**

**Authors:** **Authors:** Anna Elias-Warren^1,2^, Julia C. Bennett^1,2^, Chidozie D. Iwu^1,2^, Lea M. Starita^3,4^, Jeremy Stone^3^, Ben Capodanno^3^, Robin Prentice^3^, Peter D. Han^3,4^, Zack Acker^3^, Sally B. Grindstaff^3^, David Reinhart^3^, Jennifer K. Logue^1^, Caitlin R. Wolf^1^, Michael Boeckh^1,3,5^, Kevin Kong ^6^, Hong Xie ^6^, Geon Kim ^6^, Alexander L. Greninger ^6^, Amanda C. Perofsky^3,7^, Cécile Viboud^7^, Timothy M. Uyeki^9^,  Janet A. Englund^10^, Pavitra Roychoudhury^5,6^, Helen Y. Chu^1^

**Author Affiliations:**

^1^Department of Medicine, University of Washington, Seattle, WA, USA

^2^Department of Epidemiology, University of Washington, Seattle, WA, USA

^3^Brotman Baty Institute for Precision Medicine, Seattle, WA, USA

^4^Department of Genome Sciences, University of Washington, Seattle, WA, USA

^5^Vaccine and Infectious Disease Division, Fred Hutchinson Cancer Center, Seattle, WA, USA

^6^Department of Laboratory Medicine and Pathology, University of Washington, Seattle, WA, USA

^7^Fogarty International Center, National Institutes of Health, Bethesda, MD, USA

^8^Howard Hughes Medical Institute, Seattle, WA, USA

^9^Centers for Disease Control and Prevention, Atlanta, GA, USA

^10^Seattle Children’s Research Institute, Seattle, WA, USA

***Correspondence:**

E-mail Address: Anna Elias-Warren; annaew@uw.edu

Postal Address: UW Medicine at South Lake Union

Chu Lab Room E600

750 Republican St.

Seattle WA, 98109

**Supplementary Material**

Community Sampling

After the onset of the pandemic, in-person kiosks were closed and “swab-and-send" was continued in partnership with Public Health Seattle King County as the Seattle Coronavirus Assessment Network (SCAN) substudy.13

**Figure 1. Seattle Flu Study community-based respiratory virus surveillance study timelines and nasal swab collection methods**

| **SFS Substudy** | | **2018** | | **2019** | | **2020** | | | **2021** | **2022** | |
| --- | --- | --- | --- | --- | --- | --- | --- | --- | --- | --- | --- |
| Kiosks | |  | |  | |  | | |  |  | |
| Swab and Send | |  | |  | |  | | |  |  | |
| SCAN^1^ | |  | |  | |  | | |  |  | |
|  | **Swabs:** | |  | |  | |  |  | | | |
| \|  \| Collected by research staff \| \| --- \| --- \| \|  \| Self-collected & observed by study staff (as a safety measure following implementation of COVID-19 precautions) \| \|  \| Self-collected at home (unobserved) & returned to the laboratory by mail \| | | | | | | | | | | |  |

^1^Seattle Coronavirus Assessment Network

Sequencing Methods

RNA was extracted using the MagnaPure 96 DNA and viral nucleic acid small volume kit (Roche Diagnostics), with 200μL input and 50μL elution. Extracted RNA was converted to double-stranded cDNA, purified by bead cleanup, enzymatically fragmented, end-repaired, indexed, amplified, and purified again using the QIAseq FX DNA Library Kit (Qiagen). Hybridization capture was performed using the QIAseq xHYB Viral Respiratory Panel (Qiagen) after pooling libraries by sample Crt values, with four samples in each pool. After overnight hybridization with biotinylated probes and subsequent washing to remove unbound fragments, enriched libraries were amplified and then purified by bead clean-up. Library fragment sizes were estimated by TapeStation 4200 D1000 (Agilent) and concentrations were measured by Qubit 4 Fluorometer (Invitrogen). Libraries passing quality control were sequenced on Illumina Novaseq 6000 or Nextseq 2000 instruments using a 2x150 read format. Consensus genomes were generated by using a custom bioinformatic pipeline (<https://github.com/greninger-lab/revica>) described previously. This pipeline performs trimming of raw reads for quality, reference selection, and iterative mapping to generate a consensus genome. Sequencing data has been uploaded to NCBI BioProject PRJNA1029161 (Accessions available in Supplementary Table 1).

**Supplementary Table 1**. Study sequence data deposited to NCBI GenBank (Bioproject PRJNA1029161)

| sequence name | collection_date | genbank accession |
| --- | --- | --- |
| WA-UW-8fd40 | 28-May-19 | PP947665 |
| WA-UW-f73d6 | 26-Apr-19 | PP947573 |
| WA-UW-9d66c | 14-Mar-19 | PP947654 |
| WA-UW-0e9b8 | 2-Jan-20 | PP947619 |
| WA-UW-e9358 | 28-Jan-20 | PP947558 |
| WA-UW-63949 | 7-Mar-20 | PP947642 |
| WA-UW-e2d22 | 3-May-19 | PP947700 |
| WA-UW-443b4 | 24-Feb-20 | PP947688 |
| WA-UW-12020 | 3-Jun-19 | PP947658 |
| WA-UW-ef288 | 13-Mar-19 | PP947616 |
| WA-UW-eb6d0 | 21-Feb-20 | PP947602 |
| WA-UW-0d1ac | 12-Mar-20 | PP947599 |
| WA-UW-801c4 | 5-Mar-20 | PP947570 |
| WA-UW-bf75d | 30-Nov-19 | PP947682 |
| WA-UW-baa93 | 26-Feb-20 | PP947679 |
| WA-UW-9e248 | 3-Mar-20 | PP947696 |
| WA-UW-6e860 | 22-Jan-20 | PP947635 |
| WA-UW-51ee8 | 7-Mar-20 | PP947563 |
| WA-UW-03d75 | 8-Jan-20 | PP947676 |
| WA-UW-80ded | 14-Mar-20 | PP947650 |
| WA-UW-972ba | 1-Apr-20 | PP947641 |
| WA-UW-49dc2 | 23-Feb-19 | PP947662 |
| WA-UW-8126f | 30-Dec-18 | PP947652 |
| WA-UW-9efbe | 24-Feb-20 | PP947655 |
| WA-UW-8f015 | 26-Jan-19 | PP947587 |
| WA-UW-c9df8 | 12-Mar-19 | PP947697 |
| WA-UW-72558 | 30-Mar-20 | PP947601 |
| WA-UW-6df07 | 4-Mar-20 | PP947608 |
| WA-UW-4e433 | 8-Jan-20 | PP947699 |
| WA-UW-1188c | 28-Feb-20 | PP947636 |
| WA-UW-dfafa | 9-Mar-20 | PP947559 |
| WA-UW-ee060 | 17-Mar-19 | PP947653 |
| WA-UW-0ce81 | 5-Mar-20 | PP947564 |
| WA-UW-63a02 | 6-Apr-20 | PP947577 |
| WA-UW-90436 | 14-May-19 | PP947575 |
| WA-UW-924b3 | 10-Mar-19 | PP947624 |
| WA-UW-80ab8 | 24-Dec-19 | PP947615 |
| WA-UW-96ea2 | 14-Dec-19 | PP947611 |
| WA-UW-875db | 15-Dec-19 | PP947670 |
| WA-UW-4c273 | 20-Jul-19 | PP947593 |
| WA-UW-6e131 | 30-Nov-19 | PP947683 |
| WA-UW-30093 | 27-Feb-20 | PP947627 |
| WA-UW-39c0b | 26-Feb-20 | PP947606 |
| WA-UW-dd74e | 3-Jan-20 | PP947659 |
| WA-UW-365d7 | 26-Feb-20 | PP947666 |
| WA-UW-6ca2f | 12-Apr-19 | PP947646 |
| WA-UW-40550 | 16-Mar-20 | PP947657 |
| WA-UW-8d9f4 | 23-Dec-19 | PP947566 |
| WA-UW-30401 | 22-Jan-20 | PP947648 |
| WA-UW-9bcba | 4-Feb-20 | PP947687 |
| WA-UW-a7b41 | 6-Jan-20 | PP947674 |
| WA-UW-ab12d | 18-Jan-22 | PP947664 |
| WA-UW-d8567 | 13-Oct-19 | PP947626 |
| WA-UW-7d5fe | 13-May-19 | PP947600 |
| WA-UW-2ec7d | 7-Mar-20 | PP947625 |
| WA-UW-c360f | 30-Dec-19 | PP947649 |
| WA-UW-73b92 | 13-Apr-19 | PP947695 |
| WA-UW-a931f | 19-Mar-19 | PP947621 |
| WA-UW-460ae | 6-Mar-20 | PP947661 |
| WA-UW-5309d | 13-Mar-20 | PP947673 |
| WA-UW-950c2 | 8-Mar-20 | PP947568 |
| WA-UW-9d946 | 10-Feb-20 | PP947553 |
| WA-UW-1f19d | 13-Feb-20 | PP947579 |
| WA-UW-ff5d9 | 6-Mar-20 | PP947589 |
| WA-UW-3f489 | 29-Dec-19 | PP947562 |
| WA-UW-ef016 | 5-Mar-20 | PP947607 |
| WA-UW-bcf19 | 28-Dec-19 | PP947628 |
| WA-UW-c4799 | 28-Jan-20 | PP947612 |
| WA-UW-12534 | 13-Feb-20 | PP947668 |
| WA-UW-6faa8 | 22-Jan-19 | PP947675 |
| WA-UW-6ccf2 | 29-Jan-20 | PP947681 |
| WA-UW-a1b87 | 28-Jan-20 | PP947651 |
| WA-UW-10d7b | 7-Jan-20 | PP947647 |
| WA-UW-fc253 | 28-Jan-20 | PP947644 |
| WA-UW-068ca | 1-May-19 | PP947686 |
| WA-UW-40e0d | 27-Nov-19 | PP947588 |
| WA-UW-47ec5 | 23-Jan-20 | PP947560 |
| WA-UW-01406 | 15-Feb-20 | PP947694 |
| WA-UW-180da | 31-Mar-19 | PP947638 |
| WA-UW-822b1 | 2-May-19 | PP947594 |
| WA-UW-a5d7c | 27-Feb-19 | PP947622 |
| WA-UW-4c5e2 | 8-Jan-20 | PP947557 |
| WA-UW-9112f | 20-Jan-19 | PP947671 |
| WA-UW-a08f4 | 16-Feb-20 | PP947634 |
| WA-UW-137bb | 12-May-19 | PP947598 |
| WA-UW-4ac5d | 23-Mar-20 | PP947690 |
| WA-UW-4059d | 13-Dec-19 | PP947663 |
| WA-UW-9f11d | 17-Feb-20 | PP947610 |
| WA-UW-38dde | 23-Feb-20 | PP947572 |
| WA-UW-083ee | 15-May-19 | PP947561 |
| WA-UW-370d3 | 17-Feb-20 | PP947633 |
| WA-UW-15568 | 10-Feb-20 | PP947555 |
| WA-UW-3c7b0 | 14-Dec-19 | PP947637 |
| WA-UW-f4790 | 9-Mar-20 | PP947692 |
| WA-UW-9502b | 1-Jan-20 | PP947669 |
| WA-UW-0035b | 9-Dec-19 | PP947576 |
| WA-UW-e98fd | 5-Apr-20 | PP947585 |
| WA-UW-e7dd0 | 4-Mar-20 | PP947660 |
| WA-UW-74872 | 18-Feb-20 | PP947645 |
| WA-UW-edbec | 29-Mar-20 | PP947631 |
| WA-UW-186fa | 4-Mar-20 | PP947567 |
| WA-UW-87f30 | 26-Feb-20 | PP947643 |
| WA-UW-cf0ec | 12-Mar-19 | PP947640 |
| WA-UW-b73ba | 4-Mar-20 | PP947581 |
| WA-UW-c6c9c | 27-Dec-19 | PP947578 |
| WA-UW-6cd05 | 23-Feb-20 | PP947689 |
| WA-UW-d3f27 | 1-Jan-20 | PP947698 |
| WA-UW-2a4eb | 1-Mar-20 | PP947556 |
| WA-UW-9b009 | 2-Nov-19 | PP947630 |
| WA-UW-054da | 26-Feb-19 | PP947580 |
| WA-UW-bbc8f | 14-Apr-19 | PP947582 |
| WA-UW-2c2b1 | 20-Dec-19 | PP947685 |
| WA-UW-2e630 | 24-Jun-19 | PP947656 |
| WA-UW-2e9c8 | 14-Dec-19 | PP947591 |
| WA-UW-11f8d | 27-Feb-20 | PP947590 |
| WA-UW-55864 | 16-Feb-20 | PP947614 |
| WA-UW-ada21 | 26-Feb-20 | PP947574 |
| WA-UW-439b5 | 29-Jan-20 | PP947629 |
| WA-UW-60f1b | 1-Sep-19 | PP947584 |
| WA-UW-57687 | 25-Feb-20 | PP947565 |
| WA-UW-7e1a4 | 10-Mar-20 | PP947693 |
| WA-UW-00138 | 12-Feb-20 | PP947592 |
| WA-UW-4d448 | 4-Jan-20 | PP947667 |
| WA-UW-575a0 | 22-Mar-19 | PP947571 |
| WA-UW-add41 | 7-Jan-20 | PP947569 |
| WA-UW-d47df | 25-Dec-19 | PP947702 |
| WA-UW-5bd9c | 26-Mar-20 | PP947586 |
| WA-UW-1845e | 23-Dec-19 | PP947678 |
| WA-UW-48ff3 | 4-Dec-19 | PP947583 |
| WA-UW-6d33c | 29-Apr-22 | PP947639 |
| WA-UW-9aa8a | 22-Dec-19 | PP947623 |
| WA-UW-e1338 | 9-Jan-20 | PP947605 |
| WA-UW-98203 | 23-Mar-20 | PP947618 |
| WA-UW-26754 | 26-Jun-21 | PP947632 |
| WA-UW-7e875 | 11-Feb-20 | PP947672 |
| WA-UW-3e24e | 7-Jan-19 | PP947620 |
| WA-UW-703fb | 12-Jan-20 | PP947680 |
| WA-UW-ff64b | 1-Mar-20 | PP947613 |
| WA-UW-62ede | 7-Jun-19 | PP947554 |
| WA-UW-48c2c | 27-Dec-19 | PP947703 |
| WA-UW-57b6d | 9-Jan-20 | PP947684 |
| WA-UW-7f700 | 27-Jan-20 | PP947603 |
| WA-UW-7eac0 | 14-Dec-19 | PP947596 |
| WA-UW-69a2c | 1-Jan-20 | PP947691 |
| WA-UW-6f610 | 10-Sep-19 | PP947609 |
| WA-UW-4df06 | 23-Nov-19 | PP947604 |
| WA-UW-2e65e | 21-May-19 | PP947677 |
| WA-UW-6ec02 | 4-Feb-20 | PP947701 |
| WA-UW-b16e5 | 29-Feb-20 | PP947595 |
| WA-UW-7b6a8 | 19-Dec-19 | PP947597 |
| WA-UW-40624 | 14-Feb-19 | PP947617 |

**Supplementary** **Table 2:** Two proportion z-test of community symptom prevalence by low relative cycle threshold (Crt) value (<15) versus high Crt value ($\geq15$)

|  | High Crt (n = 116)  n, % | Low Crt (n = 51)  n, % | p-value (alpha = 0.05) |
| --- | --- | --- | --- |
| Cough | 99 (85) | 45 (88) | 0.80 |
| Rhinorrhea | 98 (84) | 42 (82) | 0.91 |
| Fatigue | 73 (63) | 31 (61) | 0.93 |
| Fever | 55 (47) | 25 (49) | 0.98 |
| Sore Throat | 71 (61) | 30 (59) | 0.91 |
| Body Aches | 43 (37) | 23 (45) | 0.42 |
| Trouble Breathing | 37 (32) | 15 (29) | 0.89 |
| Headaches | 46 (40) | 24 (47) | 0.47 |
| Sweats | 33 (28) | 12 (24) | 0.64 |
| Chills | 32 (28) | 13 (26) | 0.93 |
| Ear Pain | 19 (16) | 10 (20) | 0.78 |
| Other | 29 (25) | 10 (20) | 0.58 |

**Supplementary** **Table 3:** Community hMPV Positive Reported Symptom Frequency by Age Group in Years

|  | **0-4**  **(n = 28)**  **n (%)** | **5-17**  **(n = 12)**  **n (%)** | **18-49**  **(n = 96)**  **n (%)** | **50+**  **(n = 20)**  **n (%)** | **Overall**  **(n = 156)**  **n (%)** |
| --- | --- | --- | --- | --- | --- |
| **Cough** | 25 (89) | 11 (92) | 90 (94) | 18 (90) | 144 (92) |
| **Rhinorrhea** | 24 (86) | 10 (83) | 89 (93) | 17 (85) | 140 (90) |
| **Fatigue** | 12 (43) | 4 (33) | 72 (75) | 16 (80) | 104 (67) |
| **Fever** | 18 (64) | 5 (42) | 48 (50) | 9 (45) | 80 (51) |
| **Sore Throat** | 5 (18) | 6 (50) | 77 (80) | 13 (65) | 101 (65) |
| **Body Aches** | 1 (4) | 3 (25) | 51 (53) | 11 (55) | 66 (42) |
| **Trouble Breathing** | 3 (11) | 2 (17) | 39 (41) | 8 (40) | 52 (33) |
| **Headaches** | 1 (4) | 4 (33) | 51 (53) | 14 (70) | 70 (45) |
| **Sweats** | 0 (0) | 2 (17) | 37 (39) | 6 (30) | 45 (29) |
| **Chills** | 0 (0) | 3 (25) | 35 (36) | 7 (35) | 45 (29) |
| **Ear Pain** | 1 (4) | 0 (0) | 21 (22) | 7 (35) | 29 (19) |
| **Other** | 4 (14) | 4 (33) | 22 (23) | 9 (45) | 39 (25) |

**Supplementary** **Table 4.** Mean pairwise sequence identity for F gene computed from sequence alignments across all subtypes and for each subtype

|  | n | % identity (nt) | % identity (aa) |
| --- | --- | --- | --- |
| All subtypes | 209 | 90.76 | 96.76 |
| A2 | 75 | 99.26 | 99.85 |
| B1 | 62 | 99.21 | 99.86 |
| B2 | 72 | 99.13 | 99.69 |

**Supplementary Table 5.** Amino acid variation across different hMPV subtypes at positions within F in various functional domains

| Region | Position | A2  (n = 75) | B1  (n = 62) | B2  (n = 72) |
| --- | --- | --- | --- | --- |
| Signal Peptide | 6 | V/A | M/V | M |
|  | 7 | I | I | I/V |
|  | 9 | F | I | I |
|  | 10 | S/A | S | S |
|  | 13 | I | I/V | I |
| F2 subunit | 31 | I | I/V | I |
|  | 36 | L | L | L/H |
|  | 61 | A | T | T |
|  | 76 | S/N | S | S |
| Fusion peptide | 122 | V | I | I |
| Heptad repeat A | 135 | T | N | N |
|  | 139 | N/S | G | G |
|  | 142 | K | K | K/R |
|  | 143 | K | Q | T |
|  | 163 | R | R | R/K |
|  | 167 | D | E | E |
|  | 171 | K/R | K | K/R |
| F1 subunit | 175 | R | S | S / N |
|  | 179 | K | R / K | K / R |
|  | 184 | D | A | A |
|  | 191 | I/V | V | V |
|  | 223 | T | T | N / T |
|  | 229 | R | R | R / K |
|  | 233 | N | Y | Y |
|  | 280 | D | D / G | N / D |
|  | 286 | V | I | I |
|  | 288 | A | A / T | A |
|  | 296 | K | N | D / N |
|  | 312 | Q | K | K |
|  | 348 | K | R | R |
|  | 392 | I | I | T / I |
|  | 396 | R | R | Q / R |
|  | 404 | N | P | P |
|  | 415 | A | A | A / T |
|  | 435 | H | H / Y | H |
|  | 436 | V | V | V / I |
|  | 444 | S | S / N | N / S |
|  | 449 | V | I | I |
|  | 450 | K | R / K | R / K |
| Heptad repeat B | 466 | N | S | S |
|  | 472 | A | A | A / T |
|  | 475 | D | E | D |
|  | 479 | R | K | K / R |
|  | 482 | S / N | N | N |
| Transmembrane | 492 | I / T | I | I |
|  | 498 | I | V | I |
|  | 500 | V / I | V | V |
|  | 503 | S / F | L | L |
|  | 504 | S | T | T |
|  | 505 | M | M | M / I |
|  | 507 | L | S / L | S |
|  | 510 | I / V | I | I |
|  | 511 | F | I | I |
|  | 512 | I | I / V | I |
| Cytoplasmic tail | 517 | T / I | T | T / A |
|  | 518 | R / K | R | R / K |
|  | 520 | P / T | P | P |
|  | 521 | T | T / A / I | A / T |
|  | 522 | G / E | G | G |
|  | 523 | A / V | A | A |
|  | 525 | P | P | P / L |
|  | 528 | S | N | N |
|  | 533 | N | G | G |
|  | 538 | H | H / Y | H |
|  | 539 | S / N | S | S |

**Supplementary Table 6**. Counts for hMPV Percent Positivity Graphs

|  | | Clinical | | | Community | | |
| --- | --- | --- | --- | --- | --- | --- | --- |
| Season | GEOID10 (PUMA) | Positive tests | Total test | Percent positivity | Positive tests | Total test | Percent positivity |
| ’18-19 | 5311601 | 30 | 600 | 5.0 | 9 | 122 | 7.4 |
|  | 5311602 | 28 | 703 | 4.0 | 15 | 466 | 3.2 |
|  | 5311603 | 24 | 494 | 4.9 | 6 | 148 | 4.1 |
|  | 5311604 | 25 | 500 | 5.0 | 0 | 79 | 0 |
|  | 5311605 | 36 | 524 | 6.9 | 2 | 76 | 2.6 |
| ’19-20 | 5311601 | 17 | 474 | 3.6 | 10 | 697 | 1.4 |
|  | 5311602 | 25 | 515 | 4.9 | 20 | 765 | 2.6 |
|  | 5311603 | 8 | 279 | 2.9 | 7 | 813 | 0.9 |
|  | 5311604 | 11 | 254 | 4.3 | 10 | 568 | 1.8 |
|  | 5311605 | 15 | 341 | 4.4 | 11 | 491 | 2.2 |
| ’20-21 | 5311601 | 0 | 270 | 0 | 1 | 1490 | 0.1 |
|  | 5311602 | 0 | 258 | 0 | 1 | 1151 | 0.1 |
|  | 5311603 | 0 | 168 | 0 | 2 | 1203 | 0.2 |
|  | 5311604 | 0 | 128 | 0 | 1 | 923 | 0.1 |
|  | 5311605 | 0 | 145 | 0 | 0 | 1237 | 0 |
| ’21-22 | 5311601 | 8 | 255 | 3.1 | 2 | 225 | 0.9 |
|  | 5311602 | 10 | 258 | 3.9 | 3 | 155 | 1.9 |
|  | 5311603 | 3 | 250 | 1.2 | 4 | 211 | 1.9 |
|  | 5311604 | 7 | 230 | 3.0 | 1 | 167 | 0.6 |
|  | 5311605 | 4 | 241 | 1.7 | 3 | 223 | 1.3 |

**Supplementary Table 7**. Counts for hMPV Percent Positivity Graphs ($\geq$18 years of age)

|  | | Clinical | | | Community | | |
| --- | --- | --- | --- | --- | --- | --- | --- |
| Season | GEOID10 (PUMA) | Positive tests | Total test | Percent positivity | Positive tests | Total test | Percent positivity |
| ’18-19 | 5311601 | 12 | 283 | 4.2 | 3 | 91 | 3.3 |
|  | 5311602 | 10 | 328 | 3.0 | 9 | 438 | 2.1 |
|  | 5311603 | 18 | 251 | 5.1 | 5 | 138 | 3.6 |
|  | 5311604 | 8 | 205 | 3.9 | 0 | 68 | 0 |
|  | 5311605 | 11 | 188 | 5.9 | 1 | 64 | 1.6 |
| ’19-20 | 5311601 | 8 | 206 | 3.9 | 10 | 647 | 1.5 |
|  | 5311602 | 6 | 227 | 2.6 | 19 | 699 | 2.7 |
|  | 5311603 | 4 | 147 | 2.7 | 7 | 791 | 0.9 |
|  | 5311604 | 6 | 92 | 6.5 | 9 | 521 | 1.7 |
|  | 5311605 | 6 | 89 | 6.7 | 8 | 441 | 1.8 |
| ’20-21 | 5311601 | 0 | 22 | 0 | 0 | 1230 | 0 |
|  | 5311602 | 0 | 23 | 0 | 1 | 880 | 0.1 |
|  | 5311603 | 0 | 70 | 0 | 1 | 1002 | 0.1 |
|  | 5311604 | 0 | 32 | 0 | 0 | 760 | 0 |
|  | 5311605 | 0 | 21 | 0 | 0 | 1057 | 0 |
| ’21-22 | 5311601 | 2 | 30 | 6.7 | 1 | 179 | 0.6 |
|  | 5311602 | 1 | 34 | 2.9 | 1 | 121 | 0.8 |
|  | 5311603 | 1 | 124 | 0.8 | 1 | 183 | 0.5 |
|  | 5311604 | 0 | 85 | 0 | 1 | 144 | 0.7 |
|  | 5311605 | 0 | 70 | 0 | 3 | 166 | 1.8 |

**Supplementary Table 8**. Counts for hMPV Percent Positivity Graphs (<18 years of age)

|  | | Clinical | | | Community | | |
| --- | --- | --- | --- | --- | --- | --- | --- |
| Season | GEOID10 (PUMA) | Positive tests | Total test | Percent positivity | Positive tests | Total test | Percent positivity |
| ’18-19 | 5311601 | 18 | 317 | 5.7 | 6 | 31 | 19.3 |
|  | 5311602 | 18 | 375 | 4.8 | 6 | 28 | 21.4 |
|  | 5311603 | 6 | 143 | 4.2 | 1 | 9 | 11.1 |
|  | 5311604 | 17 | 295 | 5.8 | 0 | 11 | 0 |
|  | 5311605 | 25 | 336 | 7.4 | 1 | 12 | 8.3 |
| ’19-20 | 5311601 | 9 | 268 | 3.4 | 0 | 50 | 0 |
|  | 5311602 | 19 | 288 | 6.6 | 1 | 64 | 1.6 |
|  | 5311603 | 4 | 132 | 3.0 | 0 | 21 | 0 |
|  | 5311604 | 5 | 162 | 3.1 | 1 | 44 | 2.3 |
|  | 5311605 | 9 | 252 | 3.6 | 0 | 41 | 0 |
| ’20-21 | 5311601 | 0 | 248 | 0 | 0 | 229 | 0 |
|  | 5311602 | 0 | 235 | 0 | 0 | 225 | 0 |
|  | 5311603 | 0 | 98 | 0 | 0 | 170 | 0 |
|  | 5311604 | 0 | 96 | 0 | 0 | 150 | 0 |
|  | 5311605 | 0 | 114 | 0 | 0 | 158 | 0 |
| ’21-22 | 5311601 | 6 | 225 | 2.7 | 1 | 46 | 2.2 |
|  | 5311602 | 9 | 223 | 4.0 | 2 | 34 | 5.9 |
|  | 5311603 | 2 | 126 | 1.6 | 3 | 28 | 10.7 |
|  | 5311604 | 7 | 145 | 4.8 | 0 | 23 | 0 |
|  | 5311605 | 4 | 171 | 2.3 | 0 | 57 | 0 |

**Supplementary Table 9**. Distribution of Enrollment Date and Symptom Onset Date for Community Positives

| Days between Enrollment and Symptom Onset Date | Community Enrollments  n (%) |
| --- | --- |
| 0 | 16 (16) |
| 1 | 8 (8) |
| 2 | 19 (19) |
| 3 | 30 (30) |
| 4 | 13 (13) |
| 5 | 6 (6) |
| 6 | 5 (5) |
| 7 | 2 (2) |

**Supplementary Table 10**. Demographic characteristics of individuals tested for human metapneumovirus (hMPV) in clinical settings (N = 33,436)

**
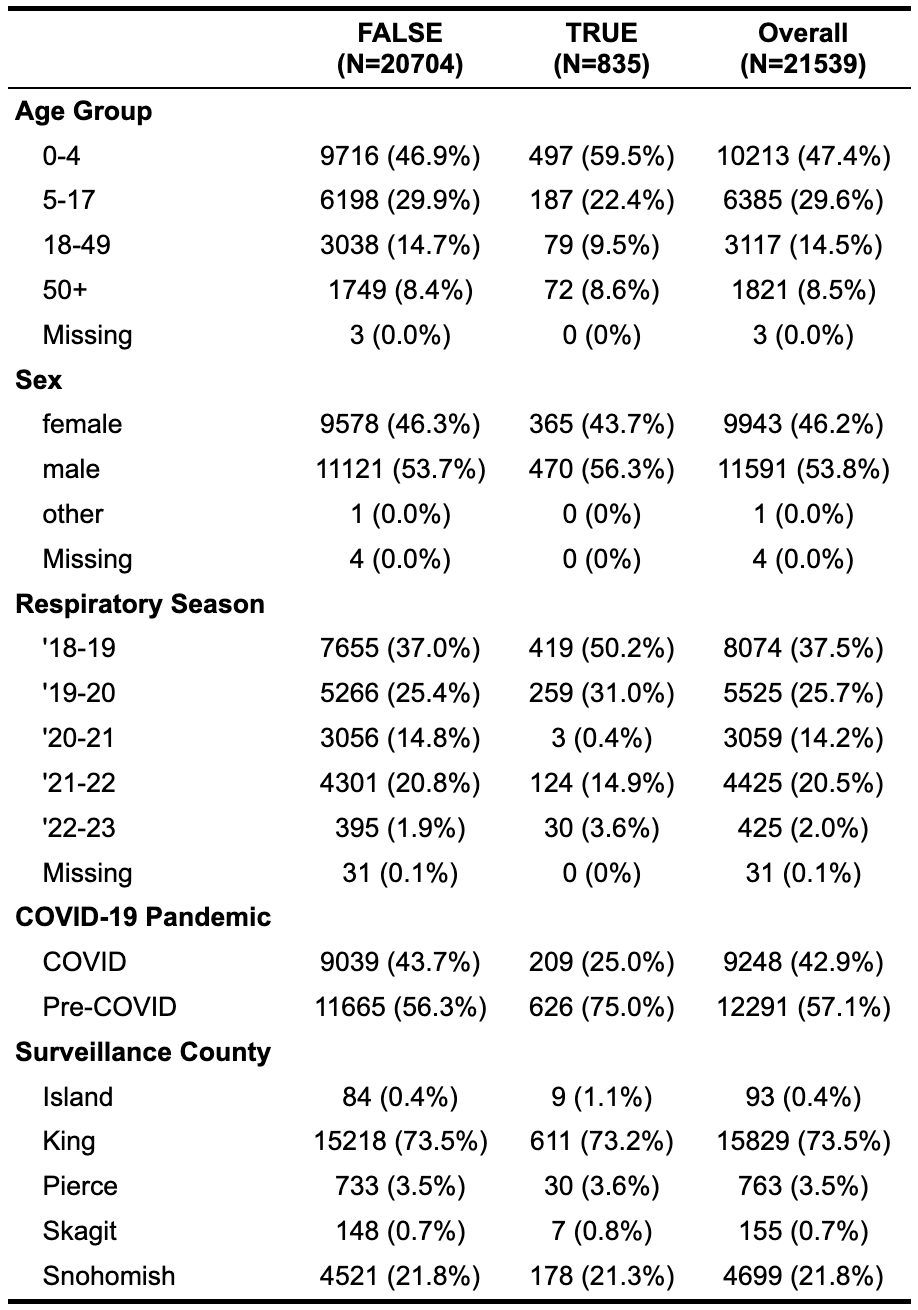
**

***Notes:*** *Anything collected on March 1^st^, 2020 or after was considered collected after the start of the pandemic. Respiratory seasons were defined as starting in July and finishing in June of the following year. County variable was created based on census tract. Community samples with a census outside of the surveillance counties were assumed to spend enough time in the surveillance counties to be included in analysis.*
